# Reporting funding source or conflict of interest in abstracts of randomized controlled trials, no evidence of a large impact on general practitioners’ confidence in conclusions, a three-arm randomized controlled trial

**DOI:** 10.1186/1741-7015-12-69

**Published:** 2014-04-28

**Authors:** Céline Buffel du Vaure, Isabelle Boutron, Elodie Perrodeau, Philippe Ravaud

**Affiliations:** 1 METHODS Team, Epidemiology and Statistics Sorbonne Paris Cité Research Centre UMR 1153, INSERM, Paris, France; 2 Université Paris Descartes, Faculté de Médecine, Paris, France; 3 Département de Médecine Générale, Université Paris Descartes, Paris, France; 4 AP-HP (Assistance Publique des Hôpitaux de Paris), Hôpital Hôtel Dieu, Centre d’Epidémiologie Clinique, Paris, France; 5 French Cochrane Centre, Paris, France; 6 Department of Epidemiology, Columbia University Mailman School of Public Health, New York, NY, USA

**Keywords:** Funding, Conflict of interest, General Practitioner, Abstract, Reporting

## Abstract

**Background:**

Systematic reporting of funding sources is recommended in the CONSORT Statement for abstracts. However, no specific recommendation is related to the reporting of conflicts of interest (CoI). The objective was to compare physicians’ confidence in the conclusions of abstracts of randomized controlled trials of pharmaceutical treatment indexed in PubMed.

**Methods:**

We planned a three-arm parallel-group randomized trial. French general practitioners (GPs) were invited to participate and were blinded to the study’s aim. We used a representative sample of 75 abstracts of pharmaceutical industry-funded randomized controlled trials published in 2010 and indexed in PubMed. Each abstract was standardized and reported in three formats: 1) no mention of the funding source or CoI; 2) reporting the funding source only; and 3) reporting the funding source and CoI. GPs were randomized according to a computerized randomization on a secure Internet system at a 1:1:1 ratio to assess one abstract among the three formats. The primary outcome was GPs’ confidence in the abstract conclusions (0, not at all, to 10, completely confident). The study was planned to detect a large difference with an effect size of 0.5.

**Results:**

Between October 2012 and June 2013, among 605 GPs contacted, 354 were randomized, 118 for each type of abstract. The mean difference (95% confidence interval) in GPs’ confidence in abstract findings was 0.2 (-0.6; 1.0) (*P* = 0.84) for abstracts reporting the funding source only versus no funding source or CoI; -0.4 (-1.3; 0.4) (*P* = 0.39) for abstracts reporting the funding source and CoI versus no funding source and CoI; and -0.6 (-1.5; 0.2) (*P* = 0.15) for abstracts reporting the funding source and CoI versus the funding source only.

**Conclusions:**

We found no evidence of a large impact of trial report abstracts mentioning funding sources or CoI on GPs’ confidence in the conclusions of the abstracts.

**Trial Registration:**

ClinicalTrials.gov identifier: NCT01679873

## Background

The source of funding of clinical trials and researchers’ conflicts of interest (CoI) are a major cause for concern in clinical research. Several empirical studies showed that pharmaceutical industry-funded trials more often report positive conclusions than do non-industry–funded trials [1-4]. Internal industry documents, which have become public because of litigation, have revealed how industry trials selectively report favorable results and modify the interpretation of results to favor their drug [5,6]. Similarly, the financial relationship among industry, scientific investigators and academic institutions can affect the presentation and interpretation of clinical trial results [7-11].

To deal with these issues, some initiatives have aimed to increase transparency in medical research. The American Medical Association encourages physicians to regularly update all their financial and CoI disclosures required by employers, advisory bodies and entities funding research. The Physician Payment Sunshine Act requires manufacturers of drugs, devices, biologicals or medical supplies to report annually certain payments or other transfers of value to physicians and teaching hospitals. The manufacturers’ reports will be available on a public searchable website [12]. The International Committee of Medical Journal Editors (ICMJE) recommends that all authors who submit a manuscript complete the ICMJE Uniform Disclosure Form for Potential Conflicts of Interest [13,14]. Finally, the CONSORT Statement for abstracts recommends reporting the funding source in the abstract of randomized trials and in the full-text articles [15,16]. However, despite these recommendations, the funding source and CoI are frequently inadequately reported [17-20]. For example, despite the CONSORT Statement for abstracts, the funding source is reported in less than 20% of abstracts for randomized controlled trials (RCTs) published in high-impact-factor journals [21], and author CoI are usually not reported in abstracts for RCTs.

The reporting of the trial funding source and the authors’ financial CoI could affect readers’ interpretations. Some studies showed that disclosure of the funding source or authors’ financial CoI in abstracts can lead readers to discount the results of a trial [22,23]. Recently, Kesselheim and colleagues showed that the reporting of industry funding in abstracts negatively affected readers’ interpretations independent of the trial’s quality [24]. However, most of these studies involved a small number of abstracts representing hypothetical scenarios of clinical trials evaluating a new drug [24,25]. Furthermore, these studies did not compare the effect of reporting both the trial funding source and the authors’ financial CoI.

We aimed to compare the confidence of primary-care physicians in the conclusions of a large representative sample of abstracts for RCTs of pharmaceutical treatment indexed in PubMed reported with or without mention of the funding source and/or authors’ financial CoI.

## Methods

### Trial design

We planned a three-arm parallel-group RCT with a sample of representative abstracts of RCTs of pharmaceutical treatment indexed in PubMed, each reported with 1) no mention of the funding source or authors’ financial CoI, 2) the funding source only or 3) the funding source and CoI.

We obtained ethics approval from the Institutional Review Board of Paris Descartes University, Paris, France (no. 2012-A00032-41), and the protocol is registered at ClinicalTrials.gov ( NCT01679873).

The study was funded by a grant from the Fondation pour la RechercheMédicale (EquipeEspoir de la Recherche, 2010). The funders were not involved in the study design; data collection, analysis, or interpretation; the writing of the article; or the decision to submit for publication.

### Selection of randomized controlled trials indexed in PubMed

We selected a sample of RCTs indexed in PubMed and published from 1 January 2010 to 31 December 2010 in the Core Clinical Journals (a subset of 119 widely read journals published in English, covering all specialties of clinical medicine and public-health sciences and including all major medical journals). The search strategy is detailed in Additional file 1. One researcher screened all retrieved citations on the title, abstract and the full text when necessary. The eligibility criteria were an industry-funded RCT with at least one author having a financial CoI, testing superiority, assessing pharmaceutical treatment (drugs) prescribed in a primary-care setting (defined as drugs that may be prescribed by a general practitioner (GP) or prescribed for diseases managed jointly by a specialist and a GP), with a conclusion in favor of the beneficial effect of the experimental treatment.

Exclusion criteria were investigation of nonpharmaceutical treatments (that is, medical devices, patient education), equivalence or noninferiority trials, safety trials, trials assessing different pharmacological procedures, and abstracts reporting a negative or neutral conclusion. One researcher (CB), who is an assistant professor in primary care, screened all retrieved abstracts and selected abstracts following these inclusion criteria. In cases of doubt regarding the inclusion of an abstract, a second researcher (IB) evaluated the abstract to achieve consensus.

From the selected reports, the reviewer systematically extracted the industry funding source and all authors’ financial CoI. If these data were not reported in the abstract or full-text article, the abstract was excluded.

Of the 2,797 citations screened, reports of 75 RCTs were selected [see Additional files 2 and 3]. In 29 reports (37%), all authors had financial CoI. In 62 (81%), more than 50% of the authors had financial CoI. In 58 (77%), authors who had financial CoI were industry employees and were the first, second or last author in 34 (45%) reports.

### Abstract construction

The abstracts for all selected RCTs were standardized and modified. The journal name, date and registration number at ClinicalTrials.gov were deleted. Authors' names were substituted by names randomly selected among the 200 names most common in the United Kingdom [see Additional file 4]. Treatments were referred to as ‘experimental treatment A’ or ‘experimental treatment B’. Acronyms used for the study were deleted and dates in the text were modified to avoid trial recognition. Furthermore, each abstract was translated into French. As a quality procedure, three GPs read all abstracts to ensure the quality of the translation and the relevance of the abstracts for GPs.

The abstract for each RCT was reported in three formats as follows:

– Abstract with no mention of the funding source or CoI. If the funding was reported in the original abstract, it was removed.

Abstract reporting the funding source only. A heading ‘FUNDING’ was added after the abstract conclusions. Under this heading, we reported the name of the pharmaceutical industry funding the trial.

– Abstract reporting the funding source and CoI. Two headings were added after the abstract conclusions. Under the first heading ‘FUNDING’, we reported the name of the pharmaceutical industry funding the trial. Under the second heading ‘CONFLICTS OF INTEREST,’ we reported the initials of authors who 1) were employed by the industry and 2) had a financial interest and/or other relationship with the industry (for example, fees, travel costs, stock options, link with a family member employed by the industry). The number of authors with CoI varied in the RCT reports (for 37% of abstracts, all authors had financial CoI; for 81%, more than 50% of authors had financial CoI; for 45%, the first, second or last author was employed by the pharmaceutical industry) (see examples in Table 1).

**Table 1:** Examples of abstracts assessed

**Abstract with no mention of funding source and CoI**	**Abstract reporting funding source only**	**Abstract reporting funding source and CoI**
**Efficacy and safety of experimental treatment A in combination with treatment B and treatment c in patients with mixed dyslipidemia.**	**Efficacy and safety of experimental treatment A in combination with treatment B and treatment c in patients with mixed dyslipidemia.**	**Efficacy and safety of experimental treatment A in combination with treatment B and treatment c in patients with mixed dyslipidemia.**
**AUTHORS:** Thomson MR; Cook A; Pettigrew GE; Bower G; Bishop D; Potter LM; Alyn JC	**AUTHORS:** Thomson MR; Cook A; Pettigrew GE; Bower G; Bishop D; Potter LM; Alyn JC	**AUTHORS:** Thomson MR; Cook A; Pettigrew GE; Bower G; Bishop D; Potter LM; Alyn JC
**BACKGROUND:** Treatment B and treatment C combination therapy may be insufficient to improve lipid and nonlipid parameters beyond low-density lipoprotein cholesterol (LDL-C) in patients with mixed dyslipidemia.	**BACKGROUND:** Treatment B and treatment C combination therapy may be insufficient to improve lipid and nonlipid parameters beyond low-density lipoprotein cholesterol (LDL-C) in patients with mixed dyslipidemia.	**BACKGROUND:** Treatment B and treatment C combination therapy may be insufficient to improve lipid and nonlipid parameters beyond low-density lipoprotein cholesterol (LDL-C) in patients with mixed dyslipidemia.
**METHODS:** In this phase 3, multicenter, double-blind study, a total of 543 patients with triglycerides >/=150 mg/dL and <400 mg/dL, high-density lipoprotein cholesterol (HDL-C) <40 mg/dL (<50 mg/dL for women), and LDL-C >/=130 mg/dL were randomized to 12 weeks of treatment with experimental treatment A 135 mg or placebo, each coadministered with treatment B 40 mg + treatment C 10 mg (treatment BC).	**METHODS:** In this phase 3, multicenter, double-blind study, a total of 543 patients with triglycerides >/=150 mg/dL and <400 mg/dL, high-density lipoprotein cholesterol (HDL-C) <40 mg/dL (<50 mg/dL for women), and LDL-C >/=130 mg/dL were randomized to 12 weeks of treatment with experimental treatment A 135 mg or placebo, each coadministered with treatment B 40 mg + treatment C 10 mg (treatment BC).	**METHODS:** In this phase 3, multicenter, double-blind study, a total of 543 patients with triglycerides >/=150 mg/dL and <400 mg/dL, high-density lipoprotein cholesterol (HDL-C) <40 mg/dL (<50 mg/dL for women), and LDL-C >/=130 mg/dL were randomized to 12 weeks of treatment with experimental treatment A 135 mg or placebo, each coadministered with treatment B 40 mg + treatment C 10 mg (treatment BC).
**RESULTS:** Both treatment regimens lowered LDL-C by >50%; however, experimental treatment A and treatment BC resulted in significantly (P < .001) greater improvements in HDL-C (13.0% vs 4.2%), triglycerides (-57.3% vs -39.7%), non-HDL-C (-55.6% vs -51.0%), and apoprotein B (-49.1% vs -44.7%) compared with treatment BC. Overall, adverse events were similar in the 2 treatment groups. No unexpected muscle, hepatic, or renal safety signals were identified with either treatment combination.	**RESULTS:** Both treatment regimens lowered LDL-C by >50%; however, experimental treatment A and treatment BC resulted in significantly (P < .001) greater improvements in HDL-C (13.0% vs 4.2%), triglycerides (-57.3% vs -39.7%), non-HDL-C (-55.6% vs -51.0%), and apoprotein B (-49.1% vs -44.7%) compared with treatment BC. Overall, adverse events were similar in the 2 treatment groups. No unexpected muscle, hepatic, or renal safety signals were identified with either treatment combination.	**RESULTS:** Both treatment regimens lowered LDL-C by >50%; however, experimental treatment A and treatment BC resulted in significantly (P < .001) greater improvements in HDL-C (13.0% vs 4.2%), triglycerides (-57.3% vs -39.7%), non-HDL-C (-55.6% vs -51.0%), and apoprotein B (-49.1% vs -44.7%) compared with treatment BC. Overall, adverse events were similar in the 2 treatment groups. No unexpected muscle, hepatic, or renal safety signals were identified with either treatment combination.
**CONCLUSIONS:** In patients with mixed dyslipidemia, the combination of experimental treatment A + treatment BC significantly improved lipid and nonlipid parameters compared with treatment BC and was generally well tolerated.	**CONCLUSIONS:** In patients with mixed dyslipidemia, the combination of experimental treatment A + treatment BC significantly improved lipid and nonlipid parameters compared with treatment BC and was generally well tolerated.	**CONCLUSIONS:** In patients with mixed dyslipidemia, the combination of experimental treatment A + treatment BC significantly improved lipid and nonlipid parameters compared with treatment BC and was generally well tolerated.
	**FUNDING:** Abbott.	**FUNDING:** Abbott.
		**CONFLICTS OF INTEREST:** MRT, AC and GEP declared financial interest and/or other relationships with Abbott. GB, DB, LMP and JCA are employees of Abbott.

CoI, conflicts of interest.

Consequently, we had 225 versions of the 75 abstracts selected: 75 with no mention of the funding source or CoI, 75 reporting the funding source only, and 75 reporting the funding source and CoI.

### Selection of general practitioners and recruitment procedures

Eligible participants were French GP members of a network of GPs involved in clinical research. GPs were from all over France and had part-time activity in a university general practice department as a teacher or student supervisor.

GPs were invited by email to participate in a study evaluating the interpretation of abstracts of RCTs (with three reminders) or by an investigator during the national annual medical congress for general practice. We used two different sources of recruitment for pragmatic reasons. Such a strategy is very often used in most RCTs to increase the sample size and the generalizability of the study results. The GPs were informed that their participation would involve reading only one abstract of an RCT and answering some questions about their interpretation of the abstract. They were informed that the collected data would remain anonymous and that we would inform them of the study results when available. However, they did not know that abstracts were modified and that several formats were compared. As approved by the ethics committee, participant consent was considered obtained as soon as they logged onto the survey site. If GPs agreed to participate, they had to complete a questionnaire related to their general characteristics. They were instructed to carefully read one abstract that was randomly selected for them and they had to answer questions related to their interpretation of this abstract (see Outcomes and Additional files 5 and 6).

### Randomization and blinding of general practitioners

An independent statistician created a computerized randomization list with a 1:1:1 ratio using random block sizes. The sequence was generated to have the same number of assessments for each of the three formats of a given abstract. A computer engineer uploaded the randomization list to a secure Internet system to assure allocation concealment. Participants logged onto this secure Internet system and were randomized to assess one of the 75 abstracts presented in one of the three formats.

Participants were informed that the aim of the study was to assess their interpretation of RCT abstracts, but they did not know that abstracts were modified and that several formats were compared.

### Outcomes

The primary outcome was GPs’ confidence in the abstract’s conclusions. GPs answered the following question: ‘On a scale ranging from 0 to 10, indicate your confidence in the conclusion reported,’ with 0, not at all confident, to 10, completely confident. The secondary endpoints were the perceived methodological quality of the study and the interpretation of the treatment benefit in terms of safety and efficacy. For these outcomes, GPs were asked ‘On a scale ranging from 0 to 10, what is the methodological quality of the study?’ with 0, very poor quality, to 10, excellent quality, and ‘On a scale ranging from 0 to 10, is the experimental treatment beneficial in terms of safety and efficacy?’ with 0, not at all beneficial, to 10, totally beneficial.

Details of the questionnaire are in Additional file 6.

### Sample size

With a significance level of 1.67% fixed for each of the three 2 × 2 comparisons (Bonferroni correction to maintain an overall significance level of 5%), we needed 118 evaluations for each format to demonstrate an effect size of 0.5 on the numeric scale with a power of 90% for each 2 × 2 comparison. Because we expected that each participant would read a single abstract, we needed to include 118 participants per arm (354 in total).

### Statistical analysis

Descriptive results are reported with means and standard deviations (SDs), median and interquartile ranges (Q1 to Q3) for quantitative variables and frequencies and percentages by modality for qualitative variables.

We had three main comparisons: abstracts with no mention of the funding source or CoI versus reporting the funding source only; abstracts with no mention of the funding source or CoI versus reporting the funding source and CoI; and abstracts reporting the funding source only versus reporting the funding source and CoI. Differences in primary outcome between groups were estimated by a linear model and were compared by Tukey’s honestly significant difference test to adjust for multiple testing [26]. Differences in secondary outcomes were compared in the same manner as for the primary outcome. Post hoc sensitivity analysis adjusted on the mode of recruitment were performed. All analyses involved use of R software v3.0.1 [27].

## Results

### General practitioner characteristics

**Figure 1:**
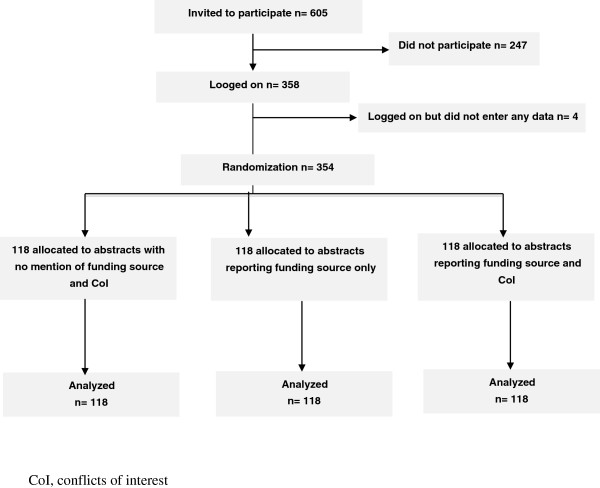
Flow diagram of general practitioner evaluators in the study.

The flow of participants is shown in Figure 1. Between October 2012 and June 2013, among 605 GPs contacted, 354 (58%) agreed to participate, and 118 were allocated to each arm. The characteristics of GPs are given in Table 2. In all, 65% were men; the median (Q1 to Q3) age was 51 (36 to 57). Less than half (43%) had participated in a pharmaceutical industry–funded clinical trial. Only 22% had received some fees from the pharmaceutical industry for speaking, consulting or enrolling patients in trials. Half had not received any visits from pharmaceutical industry representatives.

**Table 2:** General characteristics of general practitioner (GP) evaluators of abstracts of randomized controlled trials of pharmaceutical treatments

**GP characteristics**	**All GPs number = 354**	**Abstracts with no mention of funding source or CoI number = 118**	**Abstracts reporting funding source only number = 118**	**Abstracts reporting funding source and CoI number = 118**
Gender (male) - number (%)	227 (64.9)	78 (66.7)	76 (65.5)	73 (62.4)
Age, years - median (Q1 to Q3)	51.4 (36.4; 57.3)	48.7 (37.1; 56.3)	51.8 (36.2; 57.6)	52.7 (37.4; 57.2)
Receive fees from pharmaceutical industry - number (%)	75 (21.6)	30 (26.3)	20 (17.1)	25 (21.6)
For performing a presentation	22 (6.3)	8 (7.0)	7 (6.0)	7 (6.0)
For consulting	16 (4.6)	4 (3.5)	6 (5.1)	6 (5.2)
For enrolling patients in a trial	60 (17.3)	26 (22.8)	18 (15.4)	16 (13.8)
Participate (current or past) in a trial funded by pharmaceutical industry – number (%)	151 (43.5)	54 (47.4)	47 (40.2)	50 (43.1)
Receive visits from medical representatives of pharmaceutical industry - number (%)				
None	174 (50.1)	56 (49.1)	59 (50.4)	59 (50.9)
1 to 5 per month	84 (24.2)	30 (26.3)	25 (21.4)	29 (25.0)
6 to 10 per month	54 (15.6)	17 (14.9)	21 (17.9)	16 (13.8)
>10 per month	35 (10.1)	11 (9.6)	12 (10.3)	12 (10.3)

CoI, conflicts of interest.

### General practitioners’ confidence in abstract conclusions

Abstracts were read by a median of six GPs (Q1 to Q3, 3 to 6). In all, 34 abstracts were read three times (once in each group), 39 were read six times (twice in each group) and two were read nine times (three times in each group).

Data on the mean (SD) GP confidence in abstract conclusions are reported in Table 3. The mean difference (95% confidence interval (CI)) in confidence with conclusions was 0.2 (-0.6; 1.0) (*P* = 0.84) for abstracts reporting the funding source only versus no funding source or CoI; -0.4 (-1.3; 0.4) (*P* = 0.39) for abstracts reporting the funding source and CoI versus no funding source or CoI; and -0.6 (-1.5; 0.2) (*P* = 0.15) for abstracts reporting the funding source and CoI versus the funding source only (Table 3). The adjusted post-hoc analysis did not obtain exactly the same results as the analysis that was not adjusted on the mode of recruitment. However the adjusted results were very consistent and confirm that the results are robust (see Additional file 7).

**Table 3:** Comparison of GPs’ confidence in the abstract’s conclusions, methodological quality and treatment benefit

	**Abstracts with no mention of funding source or CoI number = 118**	**Abstracts reporting funding source only number = 118**	**Abstracts reporting funding source and CoI number = 118**	**Abstracts reporting funding source only versus no mention of funding source or CoI**	**Abstracts reporting funding source and CoI versus no mention of funding source or CoI**	**Abstracts reporting funding source and CoI versus funding source only**
**Outcomes**	**Mean (SD)**	**Mean (SD)**	**Mean (SD)**	**Mean difference (95%CI)**	**Mean difference (95%CI)**	**Mean difference (95%CI)**
Confidence in the abstract conclusions (scale 0 to 10)	3.6 (2.6)	3.8 (2.6)	3.2 (2.7)	0.2 (-0.6; 1.0), *P* = 0.84	-0.4 (-1.3; 0.4), *P* = 0.39	-0.6 (-1.5; 0.2), P = 0.15
Methodological quality of the study (scale 0 to 10)	4.5 (2.7)	4.6 (2.5)	4.1 (2.6)	0.1 (-0.7; 0.9), *P* = 0.97	-0.4 (-1.2; 0.4), *P* = 0.41	-0.5 (-1.3; 0.3), *P* = 0.30
Treatment benefit in terms of efficacy and safety (scale 0 to 10)	5.0 (2.8)	4.8 (2.7)	4.1 (2.8)	-0.1 (-1.0; 0.7), *P* = 0.93	-0.8 (-1.7; 0.02), *P* = 0.06	-0.7 (-1.5; 0.1), *P* = 0.13

The confidence in abstract’s conclusions is evaluated on a 0, (not at all), to 10, (completely confident) scale.

The methodological quality of the study is evaluated on a 0, (very poor), to 10, (excellent quality) scale.

Treatment benefit is evaluated on a 0, (not at all), to 10, (completely confident) scale.

Differences in outcomes between groups were estimated using a linear model and were compared with a Tukey’s HSD test [26]. CI, confidence interval; CoI, conflicts of interest; GP, general practitioner; HSD, honestly significant difference; SD, standard deviation.

### General practitioners’ perception of trial methodological quality and treatment benefit

The mean difference (95% CI) in perception of quality was 0.1 (-0.7; 0.9) (*P* = 0.97) for abstracts reporting the funding source only versus no funding source or CoI; -0.4 (-1.2; 0.4) (*P* = 0.41) for abstracts reporting the funding source and CoI versus no funding source or CoI; and -0.5 (-1.3; 0.3) (*P* = 0.30) for abstracts reporting the funding source and CoI versus the funding source only (Table 3).

The mean difference (95% CI) in assessment of treatment benefit was -0.1 (-1.0; 0.7) (*P* = 0.93) for abstracts reporting the funding source only versus no funding source or CoI; -0.8 (-1.7; 0.02) (*P* = 0.06) for abstracts reporting the funding source and CoI versus no funding source or CoI; and -0.7 (-1.5; 0.1) (*P* = 0.13) for abstracts reporting the funding source and CoI versus the funding source only (Table 3).

## Discussion

This RCT assessed the impact of reporting a funding source and/or financial CoI in trial report abstracts on GPs’ interpretation of trial results reported in the abstracts’ conclusions. We used a large representative sample of abstracts of RCTs indexed in PubMed but did not find a statistically significant difference in GPs’ confidence in the abstracts’ conclusions, assessment of trial methodological quality or perception of treatment benefit.

Although one study showed that physicians were not influenced by disclosure statements in trial reports [25], most studies evaluating how physicians interpret research funding disclosures showed that abstracts reporting a funding source or CoI could modify readers’ interpretation [22-24]. In contrast, our results did not show a statistically significant difference in GPs’ interpretation of an abstract’s findings by the disclosure statements. However, our confidence intervals were wide, with an effect going in the same direction, and confidence in abstract conclusions was surprisingly low, 3.6 on a scale from 0 to 10. Therefore, we cannot exclude an effect, particularly for abstracts reporting both a funding source and authors’ CoI.

Previous studies evaluated physicians’ interpretation of specifically developed vignettes or abstracts of hypothetical trials [22,24] or used the abstract of a single trial [23]. Contrary to these studies, we aimed to have a pragmatic approach and used abstracts from a large sample of real trials. We selected a representative sample of abstracts of RCT reports published in 2010 and indexed in PubMed, and we systematically recorded the funding source and authors’ CoI for these trials. To avoid bias, all information that could allow readers to identify the trial or drug investigated was deleted or modified by using, for example, fictitious names for authors. Abstracts reporting a negative or neutral conclusion were not included so as to homogenize abstract conclusions and facilitate the interpretation of our results.

In this trial, we evaluated the reporting of both a funding source and authors’ CoI in abstracts for several reasons. First, we focused on abstracts because they are an essential mode for disseminating research results [28-30]. Furthermore, the CONSORT statement for abstracts clearly recommends reporting the funding source to improve transparency, but we lack a clear recommendation on the reporting of authors’ financial CoI in abstracts. CoI are defined by a set of circumstances that create a risk that professional judgment or actions regarding a primary interest will be unduly influenced by a secondary interest [31]. CoI are divided into various categories, from financial to non-financial ties, such as personal relationships [32]. In our study, we did not specifically assess CoI other than financial ones. Furthermore, we explored only one factor -- reporting funding source and CoI disclosure -- and we acknowledge that in most cases, a multifaceted type of approach is required to change attitudes and behavior.

Our study has some limitations. First, in the context of a study on the interpretation of abstract results, GPs might not have interpreted abstracts as they usually do in clinical practice. Evaluation of how carefully the GPs read the abstract was not done as this is difficult to appraise. **A**nonymization of abstracts may have distanced the reader and contributed to the low confidence in the abstracts. As well, the low confidence overall suggests that GPs were more suspicious than usual, by searching details with attention. Second, these results may not be applicable to all physicians. The participants in this study were GPs involved in clinical research and/or with part-time activity in a university general practice department as a teacher or student supervisor. Our participants probably have higher expertise in clinical research than the usual French GP. Finally, this study was planned to detect a large effect, and confidence intervals were wide. Therefore, we cannot exclude a smaller effect [33], particularly for abstracts reporting both a funding source and authors’ CoI.

## Conclusions

In conclusion, we found no statistically significant difference in GPs’ interpretations of findings from abstracts reporting the funding source and/or CoI for RCTs investigating pharmaceutical treatments. However, the mean differences between sets of abstracts had wide confidence intervals and we cannot exclude a possible impact of reporting the funding source and CoI on readers’ interpretation, particularly for abstracts reporting both the funding source and authors’ financial CoI.

## Abbreviations

CI: confidence interval; CoI: conflicts of interest; GP: general practitioner; ICMJE: International Committee of Medical Journal Editors; Q: interquartile range; RCT: randomized controlled trial; SD: standard deviation.

## Competing interests

CB received honoraria from Pfizer Inc. for participating in clinical research. IB, EP and PR declare they have no conflicts of interest.

## Authors’ contributions

All authors were involved in drafting the manuscript. CB and IB were involved in acquiring and interpreting the data. EP analyzed the data. PR gave the final approval of this version of the manuscript. All authors read and approved the final manuscript.

## Pre-publication history

The pre-publication history for this paper can be accessed here:

http://www.biomedcentral.com/1741-7015/12/69/prepub

## Supplementary Material

Additional file 1 Search method. Click here for file

Additional file 2 Flow diagram of selected abstracts in the study. Click here for file

Additional file 3 References of selected abstracts. Click here for file

Additional file 4 List of sham author names used for abstracts. Click here for file

Additional file 5 Invitation to participate. Click here for file

Additional file 6 Questionnaire. Click here for file

Additional file 7 Comparison of GPs’ confidence in the abstract conclusions (primary outcome), methodological quality of the study and treatment benefit adjusted on the mode of recruitment. Click here for file
