# Supplementary material for: Reporting funding source or conflict of interest in abstracts of randomized controlled trials, no evidence of a large impact on general practitioners’ confidence in conclusions, a three-arm randomized controlled trial
Source: BMC Med. 2014 Apr 28;12:69. doi: 10.1186/1741-7015-12-69 (PMC4022327; doi:10.1186/1741-7015-12-69)
Supplement: Additional file 1 — Search method. [file 1741-7015-12-69-S1.doc]

**Additional file 1**. **Search Query in PubMed**

hasabstract[text] AND Randomized Controlled Trial[ptyp] AND English[lang] AND jsubsetaim[text] AND (“2010/01/01”[PDAT] : “2010/12/31”[PDAT])
