# Supplementary material for: Reporting funding source or conflict of interest in abstracts of randomized controlled trials, no evidence of a large impact on general practitioners’ confidence in conclusions, a three-arm randomized controlled trial
Source: BMC Med. 2014 Apr 28;12:69. doi: 10.1186/1741-7015-12-69 (PMC4022327; doi:10.1186/1741-7015-12-69)
Supplement: Additional file 2 — Flow diagram of selected abstracts in the study. [file 1741-7015-12-69-S2.doc]

**Additional file 2. Flow diagram of selected reports of randomized controlled trials (RCTs)**

PubMed search

**2,797** citations

**2,624** excluded on title and abstract, included:

- Duplicates (n=20)
- Not in field of primary care (n=809)
- Not drug intervention (n=1405)
- Equivalence, safety trial, not testing superiority (n=91)
- Reporting a negative/ambiguous conclusion (n=210)
- Not an RCT (n=46)
- Commentaries (n=3)
- Assessing different procedures (n=40)

**173** full-text articles

**98** excluded on the full-text article, included:

- Not entirely funded by industry (n= 66)
- Not reporting CoI of authors (n=18)
- Standardization not possible (n=1)
- Not in field of primary care (n=2)
- Not drug intervention (n=4)
- Equivalence trial (n=2)
- Reporting a negative/ambiguous/no conclusion (n=3)
- Not avalaible (n=2)

**75** reports of RCTs selected

CoI, conflicts of interest
