# Supplementary material for: Reporting funding source or conflict of interest in abstracts of randomized controlled trials, no evidence of a large impact on general practitioners’ confidence in conclusions, a three-arm randomized controlled trial
Source: BMC Med. 2014 Apr 28;12:69. doi: 10.1186/1741-7015-12-69 (PMC4022327; doi:10.1186/1741-7015-12-69)
Supplement: Additional file 3 — References of selected abstracts. [file 1741-7015-12-69-S3.doc]

**Additional file 3. References for the 75 selected abstracts**

1. Andriole GL, Bostwick DG, Brawley OW, Gomella LG, Marberger M, Montorsi F, Pettaway CA, Tammela TL, Teloken C, Tindall DJ, Somerville MC, Wilson TH, Fowler IL, Rittmaster RS: **Effect of dutasteride on the risk of prostate cancer.** *N Engl J Med* 2010:1192–1202.

2. Apovian CM, Bergenstal RM, Cuddihy RM, Qu Y, Lenox S, Lewis MS, Glass LC: **Effects of exenatide combined with lifestyle modification in patients with type 2 diabetes.** *Am J Med* 2010:468.e9–17.

3. Arnold LM, Gendreau RM, Palmer RH, Gendreau JF, Wang Y: **Efficacy and safety of milnacipran 100 mg/day in patients with fibromyalgia: results of a randomized, double-blind, placebo-controlled trial.** *Arthritis Rheum* 2010:2745–2756.

4. Atkinson RA, Srinivas-Shankar U, Roberts SA, Connolly MJ, Adams JE, Oldham JA, Wu FCW, Seynnes OR, Stewart CEH, Maganaris CN, Narici M V: **Effects of testosterone on skeletal muscle architecture in intermediate-frail and frail elderly men.** *J Gerontol A Biol Sci Med Sci* 2010:1215–1219.

5. Azar RR, Badaoui G, Sarkis A, Azar M, Aydanian H, Harb S, Achkouty G, Kassab R: **Effect of ezetimibe/atorvastatin combination on oxidized low density lipoprotein  cholesterol in patients with coronary artery disease or coronary artery disease equivalent.** *Am J Cardiol* 2010:193–197.

6. Bailey CJ, Gross JL, Pieters A, Bastien A, List JF: **Effect of dapagliflozin in patients with type 2 diabetes who have inadequate glycaemic control with metformin: a randomised, double-blind, placebo-controlled  trial.** *Lancet* 2010:2223–2233.

7. Bakris GL, Sarafidis PA, Weir MR, Dahlof B, Pitt B, Jamerson K, Velazquez EJ, Staikos-Byrne L, Kelly RY, Shi V, Chiang Y-T, Weber MA: **Renal outcomes with different fixed-dose combination therapies in patients with hypertension at high risk for cardiovascular events (ACCOMPLISH): a prespecified  secondary analysis of a randomised controlled trial.** *Lancet* 2010:1173–1181.

8. Bass NM, Mullen KD, Sanyal A, Poordad F, Neff G, Leevy CB, Sigal S, Sheikh MY, Beavers K, Frederick T, Teperman L, Hillebrand D, Huang S, Merchant K, Shaw A, Bortey E, Forbes WP: **Rifaximin treatment in hepatic encephalopathy.** *N Engl J Med* 2010:1071–1081.

9. Bays HE, McKenney J, Maki KC, Doyle RT, Carter RN, Stein E: **Effects of prescription omega-3-acid ethyl esters on non--high-density lipoprotein cholesterol when coadministered with escalating doses of atorvastatin.** *Mayo Clin Proc* 2010:122–128.

10. Bergenstal RM, Wysham C, Macconell L, Malloy J, Walsh B, Yan P, Wilhelm K, Malone J, Porter LE: **Efficacy and safety of exenatide once weekly versus sitagliptin or pioglitazone as an adjunct to metformin for treatment of type 2 diabetes (DURATION-2): a randomised trial.** *Lancet* 2010:431–439.

11. Bhatt DL, Cryer BL, Contant CF, Cohen M, Lanas A, Schnitzer TJ, Shook TL, Lapuerta P, Goldsmith MA, Laine L, Scirica BM, Murphy SA, Cannon CP: **Clopidogrel with or without omeprazole in coronary artery disease.** *N Engl J Med* 2010:1909–1917.

12. Bogan RK, Bornemann MAC, Kushida CA, Tran P V, Barrett RW: **Long-term maintenance treatment of restless legs syndrome with gabapentin enacarbil: a randomized controlled study.** *Mayo Clin Proc* 2010:512–521.

13. Cannon CP, Shah S, Dansky HM, Davidson M, Brinton EA, Gotto AM, Stepanavage M, Liu SX, Gibbons P, Ashraf TB, Zafarino J, Mitchel Y, Barter P: **Safety of anacetrapib in patients with or at high risk for coronary heart disease.** *N Engl J Med* 2010:2406–2415.

14. Cheng G, Saleh MN, Marcher C, Vasey S, Mayer B, Aivado M, Arning M, Stone NL, Bussel JB: **Eltrombopag for management of chronic immune thrombocytopenia (RAISE): a 6-month, randomised, phase 3 study.** *Lancet* 2011:393–402.

15. Chosidow O, Giraudeau B, Cottrell J, Izri A, Hofmann R, Mann SG, Burgess I: **Oral ivermectin versus malathion lotion for difficult-to-treat head lice.** *N Engl J Med* 2010:896–905.

16. Colombel JF, Sandborn WJ, Reinisch W, Mantzaris GJ, Kornbluth A, Rachmilewitz D, Lichtiger S, D’Haens G, Diamond RH, Broussard DL, Tang KL, van der Woude CJ, Rutgeerts P: **Infliximab, azathioprine, or combination therapy for Crohn’s disease.** *N Engl J Med* 2010:1383–1395.

17. Cosman F, Lane NE, Bolognese MA, Zanchetta JR, Garcia-Hernandez PA, Sees K, Matriano JA, Gaumer K, Daddona PE: **Effect of transdermal teriparatide administration on bone mineral density in postmenopausal women.** *J Clin Endocrinol Metab* 2010:151–158.

18. De Zeeuw D, Agarwal R, Amdahl M, Audhya P, Coyne D, Garimella T, Parving H-H, Pritchett Y, Remuzzi G, Ritz E, Andress D: **Selective vitamin D receptor activation with paricalcitol for reduction of albuminuria in patients with type 2 diabetes (VITAL study): a randomised controlled trial.** *Lancet* 2010:1543–1551.

19. Decousus H, Prandoni P, Mismetti P, Bauersachs RM, Boda Z, Brenner B, Laporte S, Matyas L, Middeldorp S, Sokurenko G, Leizorovicz A: **Fondaparinux for the treatment of superficial-vein thrombosis in the legs.** *N Engl J Med* 2010:1222–1232.

20. Diamant M, Van Gaal L, Stranks S, Northrup J, Cao D, Taylor K, Trautmann M: **Once weekly exenatide compared with insulin glargine titrated to target in patients with type 2 diabetes (DURATION-3): an open-label randomised trial.** *Lancet* 2010:2234–2243.

21. Dmochowski R, Chapple C, Nitti VW, Chancellor M, Everaert K, Thompson C, Daniell G, Zhou J, Haag-Molkenteller C: **Efficacy and safety of onabotulinumtoxinA for idiopathic overactive bladder: a double-blind, placebo controlled, randomized, dose ranging trial.** *J Urol* 2010:2416–2422.

22. Dmochowski R, Roehrborn C, Klise S, Xu L, Kaminetsky J, Kraus S: **Urodynamic effects of once daily tadalafil in men with lower urinary tract symptoms secondary to clinical benign prostatic hyperplasia: a randomized, placebo controlled 12-week clinical trial.** *J Urol* 2010:1092–1097.

23. Dohil R, Newbury R, Fox L, Bastian J, Aceves S: **Oral viscous budesonide is effective in children with eosinophilic esophagitis in a randomized, placebo-controlled trial.** *Gastroenterology* 2010:418–429.

24. Donnenfeld ED, Nichamin LD, Hardten DR, Raizman MB, Trattler W, Rajpal RK, Alpern LM, Felix C, Bradford RR, Villanueva L, Hollander DA, Schiffman RM: **Twice-daily, preservative-free ketorolac 0.45% for treatment of inflammation and  pain after cataract surgery.** *Am J Ophthalmol* 2011:420–6.e1.

25. Durham SR, Emminger W, Kapp A, Colombo G, de Monchy JGR, Rak S, Scadding GK, Andersen JS, Riis B, Dahl R: **Long-term clinical efficacy in grass pollen-induced rhinoconjunctivitis after treatment with SQ-standardized grass allergy immunotherapy tablet.** *J Allergy Clin Immunol* 2010:131–137.

26. Eid NS, Noonan MJ, Chipps B, Parasuraman B, Miller CJ, O’Brien CD: **Once- vs twice-daily budesonide/formoterol in 6- to 15-year-old patients with stable asthma.** *Pediatrics* 2010:e565–75.

27. Emery P, Breedveld F, van der Heijde D, Ferraccioli G, Dougados M, Robertson D, Pedersen R, Koenig AS, Freundlich B: **Two-year clinical and radiographic results with combination etanercept-methotrexate therapy versus monotherapy in early rheumatoid arthritis: a two-year, double-blind, randomized study.** *Arthritis Rheum* 2010:674–682.

28. Ensrud K, LaCroix A, Thompson JR, Thompson DD, Eastell R, Reid DM, Vukicevic S, Cauley J, Barrett-Connor E, Armstrong R, Welty F, Cummings S: **Lasofoxifene and cardiovascular events in postmenopausal women with osteoporosis: Five-year results from the Postmenopausal Evaluation and Risk Reduction with Lasofoxifene (PEARL) trial.** *Circulation* 2010:1716–1724.

29. Everett BM, Glynn RJ, MacFadyen JG, Ridker PM: **Rosuvastatin in the prevention of stroke among men and women with elevated levels of C-reactive protein: justification for the Use of Statins in Prevention: an Intervention Trial Evaluating Rosuvastatin (JUPITER).** *Circulation* 2010:143–150.

30. Fagerstrom K, Gilljam H, Metcalfe M, Tonstad S, Messig M: **Stopping smokeless tobacco with varenicline: randomised double blind placebo controlled trial.** *BMJ* 2010:c6549.

31. Farnier M, Ducobu J, Bryniarski L: **Efficacy and safety of adding fenofibrate 160 mg in high-risk patients with mixed hyperlipidemia not controlled by pravastatin 40 mg monotherapy.** *Am J Cardiol* 2010:787–792.

32. Fazio S, Guyton JR, Polis AB, Adewale AJ, Tomassini JE, Ryan NW, Tershakovec AM: **Long-term safety and efficacy of triple combination ezetimibe/simvastatin plus extended-release niacin in patients with hyperlipidemia.** *Am J Cardiol* 2010:487–494.

33. Gane EJ, Roberts SK, Stedman CAM, Angus PW, Ritchie B, Elston R, Ipe D, Morcos PN, Baher L, Najera I, Chu T, Lopatin U, Berrey MM, Bradford W, Laughlin M, Shulman NS, Smith PF: **Oral combination therapy with a nucleoside polymerase inhibitor (RG7128) and danoprevir for chronic hepatitis C genotype 1 infection (INFORM-1): a randomised, double-blind, placebo-controlled, dose-escalation trial.** *Lancet* 2010:1467–1475.

34. Garcia-Borreguero D, Larrosa O, Williams A-M, Albares J, Pascual M, Palacios JC, Fernandez C: **Treatment of restless legs syndrome with pregabalin: a double-blind, placebo-controlled study.** *Neurology* 2010:1897–1904.

35. Genevay S, Viatte S, Finckh A, Zufferey P, Balague F, Gabay C: **Adalimumab in severe and acute sciatica: a multicenter, randomized, double-blind, placebo-controlled trial.** *Arthritis Rheum* 2010:2339–2346.

36. Genovese MC, Van den Bosch F, Roberson SA, Bojin S, Biagini IM, Ryan P, Sloan-Lancaster J: **LY2439821, a humanized anti-interleukin-17 monoclonal antibody, in the treatment  of patients with rheumatoid arthritis: A phase I randomized, double-blind, placebo-controlled, proof-of-concept study.** *Arthritis Rheum* 2010:929–939.

37. Greenway FL, Fujioka K, Plodkowski RA, Mudaliar S, Guttadauria M, Erickson J, Kim DD, Dunayevich E: **Effect of naltrexone plus bupropion on weight loss in overweight and obese adults (COR-I): a multicentre, randomised, double-blind, placebo-controlled, phase 3 trial.** *Lancet* 2010:595–605.

38. Griffiths CEM, Strober BE, van de Kerkhof P, Ho V, Fidelus-Gort R, Yeilding N, Guzzo C, Xia Y, Zhou B, Li S, Dooley LT, Goldstein NH, Menter A: **Comparison of ustekinumab and etanercept for moderate-to-severe psoriasis.** *N Engl J Med* 2010:118–128.

39. Gruber C, van Stuijvenberg M, Mosca F, Moro G, Chirico G, Braegger CP, Riedler J, Boehm G, Wahn U: **Reduced occurrence of early atopic dermatitis because of immunoactive prebiotics  among low-atopy-risk infants.** *J Allergy Clin Immunol* 2010:791–797.

40. Hull RD, Schellong SM, Tapson VF, Monreal M, Samama M-M, Nicol P, Vicaut E, Turpie AGG, Yusen RD: **Extended-duration venous thromboembolism prophylaxis in acutely ill medical patients with recently reduced mobility: a randomized trial.** *Ann Intern Med* 2010:8–18.

41. Joffe H, Petrillo L, Viguera A, Koukopoulos A, Silver-Heilman K, Farrell A, Yu G, Silver M, Cohen LS: **Eszopiclone improves insomnia and depressive and anxious symptoms in perimenopausal and postmenopausal women with hot flashes: a randomized, double-blinded, placebo-controlled crossover trial.** *Am J Obstet Gynecol* 2010:171.e1–171.e11.

42. Johnston JM, Kurtz CB, Macdougall JE, Lavins BJ, Currie MG, Fitch DA, O’Dea C, Baird M, Lembo AJ: **Linaclotide improves abdominal pain and bowel habits in a phase IIb study of patients with irritable bowel syndrome with constipation.** *Gastroenterology* 2010:1877–1886.e2.

43. Jones PH, Goldberg AC, Knapp HR, Kelly MT, Setze CM, Stolzenbach JC, Sleep DJ: **Efficacy and safety of fenofibric acid in combination with atorvastatin and ezetimibe in patients with mixed dyslipidemia.** *Am Heart J* 2010:759–766.

44. Kaunitz AM, Bissonnette F, Monteiro I, Lukkari-Lax E, Muysers C, Jensen JT: **Levonorgestrel-releasing intrauterine system or medroxyprogesterone for heavy menstrual bleeding: a randomized controlled trial.** *Obstet Gynecol* 2010:625–632.

45. Koren G, Clark S, Hankins GD V, Caritis SN, Miodovnik M, Umans JG, Mattison DR: **Effectiveness of delayed-release doxylamine and pyridoxine for nausea and vomiting of pregnancy: a randomized placebo controlled trial.** *Am J Obstet Gynecol* 2010:571.e1–7.

46. Kovacs TO, Freston JW, Haber MM, Atkinson S, Hunt B, Peura DA: **Long-term quality of life improvement in subjects with healed erosive esophagitis: treatment with lansoprazole.** *Dig Dis Sci* 2010:1325–1336.

47. Lassen MR, Gallus A, Raskob GE, Pineo G, Chen D, Ramirez LM: **Apixaban versus enoxaparin for thromboprophylaxis after hip replacement.** *N Engl J Med* 2010:2487–2498.

48. Lembo AJ, Kurtz CB, Macdougall JE, Lavins BJ, Currie MG, Fitch DA, Jeglinski BI, Johnston JM: **Efficacy of linaclotide for patients with chronic constipation.** *Gastroenterology* 2010:886–95.e1.

49. Leonardi C, Langley RG, Papp K, Tyring SK, Wasel N, Vender R, Unnebrink K, Gupta SR, Valdecantos WC, Bagel J: **Adalimumab for treatment of moderate to severe chronic plaque psoriasis of the hands and feet: efficacy and safety results from REACH, a randomized, placebo-controlled, double-blind trial.** *Arch Dermatol* 2011:429–436.

50. Ling W, Casadonte P, Bigelow G, Kampman KM, Patkar A, Bailey GL, Rosenthal RN, Beebe KL: **Buprenorphine implants for treatment of opioid dependence: a randomized controlled trial.** *JAMA* 2010:1576–1583.

51. Lukes AS, Moore KA, Muse KN, Gersten JK, Hecht BR, Edlund M, Richter HE, Eder SE, Attia GR, Patrick DL, Rubin A, Shangold GA: **Tranexamic acid treatment for heavy menstrual bleeding: a randomized controlled trial.** *Obstet Gynecol* 2010:865–875.

52. Massanari M, Nelson H, Casale T, Busse W, Kianifard F, Geba GP, Zeldin RK: **Effect of pretreatment with omalizumab on the tolerability of specific immunotherapy in allergic asthma.** *J Allergy Clin Immunol* 2010:383–389.

53. McHutchison JG, Manns MP, Muir AJ, Terrault NA, Jacobson IM, Afdhal NH, Heathcote EJ, Zeuzem S, Reesink HW, Garg J, Bsharat M, George S, Kauffman RS, Adda N, Di Bisceglie AM: **Telaprevir for previously treated chronic HCV infection.** *N Engl J Med* 2010:1292–1303.

54. Pratley RE, Nauck M, Bailey T, Montanya E, Cuddihy R, Filetti S, Thomsen AB, Sondergaard RE, Davies M: **Liraglutide versus sitagliptin for patients with type 2 diabetes who did not have adequate glycaemic control with metformin: a 26-week, randomised, parallel-group, open-label trial.** *Lancet* 2010:1447–1456.

55. Prenner BM, Lanier BQ, Bernstein DI, Shekar T, Teper A: **Mometasone furoate nasal spray reduces the ocular symptoms of seasonal allergic rhinitis.** *J Allergy Clin Immunol* 2010:1247–1253.e5.

56. Raal FJ, Santos RD, Blom DJ, Marais AD, Charng M-J, Cromwell WC, Lachmann RH, Gaudet D, Tan JL, Chasan-Taber S, Tribble DL, Flaim JD, Crooke ST: **Mipomersen, an apolipoprotein B synthesis inhibitor, for lowering of LDL cholesterol concentrations in patients with homozygous familial hypercholesterolaemia: a randomised, double-blind, placebo-controlled trial.** *Lancet* 2010:998–1006.

57. Rosenberg RP, Bogan RK, Tiller JM, Yang R, Youakim JM, Earl CQ, Roth T: **A phase 3, double-blind, randomized, placebo-controlled study of armodafinil for  excessive sleepiness associated with jet lag disorder.** *Mayo Clin Proc* 2010:630–638.

58. Ruilope LM, Dukat A, Bohm M, Lacourciere Y, Gong J, Lefkowitz MP: **Blood-pressure reduction with LCZ696, a novel dual-acting inhibitor of the angiotensin II receptor and neprilysin: a randomised, double-blind, placebo-controlled, active comparator study.** *Lancet* 2010:1255–1266.

59. Seftel A, Goldfischer E, Kim ED, Dula E, Zeigler H, Burns P: **Onset of efficacy of tadalafil once daily in men with erectile dysfunction: a randomized, double-blind, placebo controlled trial.** *J Urol* 2011:243–248.

60. Smith MR, Morton RA, Barnette KG, Sieber PR, Malkowicz SB, Rodriguez D, Hancock ML, Steiner MS: **Toremifene to reduce fracture risk in men receiving androgen deprivation therapy  for prostate cancer.** *J Urol* 2010:1316–1321.

61. Smith SR, Weissman NJ, Anderson CM, Sanchez M, Chuang E, Stubbe S, Bays H, Shanahan WR: **Multicenter, placebo-controlled trial of lorcaserin for weight management.** *N Engl J Med* 2010:245–256.

62. So A, De Meulemeester M, Pikhlak A, Yucel AE, Richard D, Murphy V, Arulmani U, Sallstig P, Schlesinger N: **Canakinumab for the treatment of acute flares in difficult-to-treat gouty arthritis: Results of a multicenter, phase II, dose-ranging study.** *Arthritis Rheum* 2010:3064–3076.

63. Srinivas-Shankar U, Roberts SA, Connolly MJ, O’Connell MDL, Adams JE, Oldham JA, Wu FCW: **Effects of testosterone on muscle strength, physical function, body composition,  and quality of life in intermediate-frail and frail elderly men: a randomized, double-blind, placebo-controlled study.** *J Clin Endocrinol Metab* 2010:639–650.

64. Staevska M, Popov TA, Kralimarkova T, Lazarova C, Kraeva S, Popova D, Church DS, Dimitrov V, Church MK: **The effectiveness of levocetirizine and desloratadine in up to 4 times conventional doses in difficult-to-treat urticaria.** *J Allergy Clin Immunol* 2010:676–682.

65. Swedberg K, Komajda M, Bohm M, Borer JS, Ford I, Dubost-Brama A, Lerebours G, Tavazzi L: **Ivabradine and outcomes in chronic heart failure (SHIFT): a randomised placebo-controlled study.** *Lancet* 2010:875–885.

66. Tandon MK, Phillips M, Waterer G, Dunkley M, Comans P, Clancy R: **Oral immunotherapy with inactivated nontypeable Haemophilus influenzae reduces severity of acute exacerbations in severe COPD.** *Chest* 2010:805–811.

67. Tashkin DP, Rennard S, Hays JT, Ma W, Lawrence D, Lee TC: **Effects of varenicline on smoking cessation in patients with mild to moderate COPD: a randomized controlled trial.** *Chest* 2011:591–599.

68. Ten Berg JM, van ’t Hof AWJ, Dill T, Heestermans T, van Werkum JW, Mosterd A, van Houwelingen G, Koopmans PC, Stella PR, Boersma E, Hamm C: **Effect of early, pre-hospital initiation of high bolus dose tirofiban in patients with ST-segment elevation myocardial infarction on short- and long-term clinical  outcome.** *J Am Coll Cardiol* 2010:2446–2455.

69. Vaezi MF, Hagaman DD, Slaughter JC, Tanner SB, Duncavage JA, Allocco CT, Sparkman C, Clement LE, Wasden CM, Wirth D, Goutte M, McCafferty BA, Lanza DC: **Proton pump inhibitor therapy improves symptoms in postnasal drainage.** *Gastroenterology* 2010:1887–1893.e1; quiz e11.

70. Villines TC, Stanek EJ, Devine PJ, Turco M, Miller M, Weissman NJ, Griffen L, Taylor AJ: **The ARBITER 6-HALTS Trial (Arterial Biology for the Investigation of the Treatment Effects of Reducing Cholesterol 6-HDL and LDL Treatment Strategies in Atherosclerosis): final results and the impact of medication adherence, dose, and treatment duration.** *J Am Coll Cardiol* 2010:2721–2726.

71. Vogel R, Crockett RS, Oden N, Laliberte TW, Molina L: **Demonstration of efficacy in the treatment of dry eye disease with 0.18% sodium hyaluronate ophthalmic solution (vismed, rejena).** *Am J Ophthalmol* 2010:594–601.

72. Zannad F, McMurray JJ V, Krum H, van Veldhuisen DJ, Swedberg K, Shi H, Vincent J, Pocock SJ, Pitt B: **Eplerenone in patients with systolic heart failure and mild symptoms.** *N Engl J Med* 2011:11–21.

73. Zielen S, Kardos P, Madonini E: **Steroid-sparing effects with allergen-specific immunotherapy in children with asthma: a randomized controlled trial.** *J Allergy Clin Immunol* 2010:942–949.

74. Zieve F, Wenger NK, Ben-Yehuda O, Constance C, Bird S, Lee R, Hanson ME, Jones-Burton C, Tershakovec AM: **Safety and efficacy of ezetimibe added to atorvastatin versus up titration of atorvastatin to 40 mg in Patients > or = 65 years of age (from the ZETia in the ELDerly [ZETELD] study).** *Am J Cardiol* 2010:656–663.

75. Zinman B, Harris SB, Neuman J, Gerstein HC, Retnakaran RR, Raboud J, Qi Y, Hanley AJG: **Low-dose combination therapy with rosiglitazone and metformin to prevent type 2 diabetes mellitus (CANOE trial): a double-blind randomised controlled study.** *Lancet* 2010:103–111.
