# Supplementary material for: Reporting funding source or conflict of interest in abstracts of randomized controlled trials, no evidence of a large impact on general practitioners’ confidence in conclusions, a three-arm randomized controlled trial
Source: BMC Med. 2014 Apr 28;12:69. doi: 10.1186/1741-7015-12-69 (PMC4022327; doi:10.1186/1741-7015-12-69)
Supplement: Additional file 4 — List of sham author names used for abstracts. [file 1741-7015-12-69-S4.doc]

**Additional file 4.** **List of sham author names used for abstracts**

We used a sample of 20 names among a list of 200 names most common in the United Kingdom.

Selected names were: Thomson MR; Cook A; Pettigrew GE; Bower G; Bishop D; Potter LM; Alyn JC; Willy KH; Tracy A; Mac-Gregor T; Carthew ML; Curtis H; Roby S; Harry S; John S; Roberson RP; Luckin JW; Berkelay K; Crossmann B; Garrison
