# Supplementary material for: Reporting funding source or conflict of interest in abstracts of randomized controlled trials, no evidence of a large impact on general practitioners’ confidence in conclusions, a three-arm randomized controlled trial
Source: BMC Med. 2014 Apr 28;12:69. doi: 10.1186/1741-7015-12-69 (PMC4022327; doi:10.1186/1741-7015-12-69)
Supplement: Additional file 5 — Invitation to participate. [file 1741-7015-12-69-S5.doc]

**Additional file 5. Invitation to participate**

Dear colleague,

We invite you to participate to a study assessing interpretation of abstracts of randomized controlled trials. This study is being carried out by Céline Buffel du Vaure, assistant professor in primary care (Paris Descartes University).

Your participation involve reading **only 1 abstract** of an RCT and answer some questions. You will be informed of the study results when available.

**This study has nothing to do with test your methodological knowledge.**

**To praticipate Click here**

Dr Céline Buffel du Vaure et Pr Serge Gilberg (Département Médecine Générale), Pr Isabelle Boutron et Pr Philippe Ravaud (Centre Cochrane Français, INSERM U738), Université Paris Descartes, France

***Confidentiality***

Data collection remains anonymous. No data regarding your patients will be collect.
