# Supplementary material for: Reporting funding source or conflict of interest in abstracts of randomized controlled trials, no evidence of a large impact on general practitioners’ confidence in conclusions, a three-arm randomized controlled trial
Source: BMC Med. 2014 Apr 28;12:69. doi: 10.1186/1741-7015-12-69 (PMC4022327; doi:10.1186/1741-7015-12-69)
Supplement: Additional file 6 — Questionnaire. [file 1741-7015-12-69-S6.doc]

**Additional file 6.** **Questionnaire**

**Initials :** Name**: ____** Surname : ____

**Gender:** F ☐ M ☐

**Date of birth:**

Please read carefully followed abstract and answer following questions:

On a scale ranging from 0 to 10, is the **experimental treatment beneficial in terms of safety and efficacy**?

0☐ 1☐ 2☐ 3☐ 4☐ 5☐ 6☐ 7☐ 8☐ 9☐ 10☐

« not at all beneficial» « totally beneficial»

On a scale ranging from 0 to 10, what is the **methodological quality** of the study?

0☐ 1☐ 2☐ 3☐ 4☐ 5☐ 6☐ 7☐ 8☐ 9☐ 10☐

« very poor quality» « excellent quality»

On a scale ranging from 0 to 10, indicate your **confidence in the conclusion** reported?

0☐ 1☐ 2☐ 3☐ 4☐ 5☐ 6☐ 7☐ 8☐ 9☐ 10☐

« not at all confident»  «completely confident»

On a scale ranging from 0 to 10, do you believe that industry funding sources influence results of trials?

0 ☐ 1 ☐ 2 ☐ 3 ☐ 4 ☐ 5 ☐ 6 ☐ 7 ☐ 8 ☐ 9 ☐ 10 ☐

« not influence at all » « totally influence »

During the past year, did you receive from pharmaceutical industries:

Yes No

Fees for performing a presentation ☐ ☐

Fees for consulting ☐ ☐

Fees for enrolling patients in a trial ☐ ☐

Do you or did you **participate in a trial funded by pharmaceutical industry?**

☐ Yes ☐ No

How many visits from **medical representatives of pharmaceutical industry** do you receive per months?

☐ None ☐ 1 to 5 ☐ 5 to 10 ☐ more than 10
