# Supplementary material for: Reporting funding source or conflict of interest in abstracts of randomized controlled trials, no evidence of a large impact on general practitioners’ confidence in conclusions, a three-arm randomized controlled trial
Source: BMC Med. 2014 Apr 28;12:69. doi: 10.1186/1741-7015-12-69 (PMC4022327; doi:10.1186/1741-7015-12-69)
Supplement: Additional file 7 — Comparison of GPs’ confidence in the abstract conclusions (primary outcome), methodological quality of the study and treatment benefit adjusted on the mode of recruitment. [file 1741-7015-12-69-S7.doc]

**Additional file 7. Comparison of GPs’ confidence in the abstract conclusions (primary outcome), methodological quality of the study and treatment benefit adjusted on the mode of recruitment.**

|  | **Abstracts reporting**  **funding source only**  **vs**  **no mention of funding source or CoI** | **Abstracts reporting funding source and CoI**  **vs**  **no mention of funding source or CoI** | **Abstracts reporting funding source and CoI**  **vs**  **funding source only** |
| --- | --- | --- | --- |
| **Outcomes** | **Mean difference [95%CI]** | **Mean difference [95%CI]** | **Mean difference [95%CI]** |
| **Confidence in the abstract conclusions (scale 0 to 10)** | 0.2 [-0.6 ; 1.0], p=0.70 | -0.4 [-1.3 ; 0.3], p=0.27 | -0.6 [-1.4 ; 0.2], p=0.14 |
| **Methodological quality of the study (scale 0 to 10)** | 0.1 [-0.7 ; 0.9], p=0.88 | -0.4 [-1.2 ; 0.3], p=0.29 | -0.5 [-1.3 ; 0.3], p=0.23 |
| **Treatment benefit in terms of efficacy and safety (scale 0 to 10)** | -0.1 [-1.0 ; 0.7], p=0.72 | -0.8 [-1.7 ; 0.1], p=0.06 | -0.7 [-1.5 ; 0.16], p=0.11 |
